# Identification and Evaluation of African Lion (*Panthera leo*) Cub Welfare in Wildlife-Interaction Tourism

**DOI:** 10.3390/ani11092748

**Published:** 2021-09-20

**Authors:** Ann Wilson, Clive J. C. Phillips

**Affiliations:** 1 Applied Behavioural Ecology and Ecosystem Research Unit (ABEERU), Department of Agriculture and Environmental Sciences, University of South Africa, Private Bag X6, Florida 1710, South Africa; 2 Curtin University Sustainability Policy (CUSP) Institute, Curtin University, Kent St., Bentley, WA 6102, Australia; clive.phillips@curtin.edu.au

**Keywords:** animal welfare, assessing welfare, wildlife tourism, lion cub interactions, South Africa

## Abstract

**Simple Summary:**

African lion cubs are used in South Africa in wildlife-interaction tourism (cub petting). The welfare impacts on the cubs are unclear. A workshop was held with 15 lion-experienced stakeholders who all indicated a range of welfare concerns for discussion and prioritisation. The leading welfare concern identified was the ‘lack of governance and regulation’ within the industry. Participants also agreed on nine non-negotiable practices that affect cubs’ welfare. Some of these included ethical concerns, such as cubs exiting into the ranching industry (farming of lions for hunting) and the bone trade (lions being slaughtered for their bones, which are exported for lion bone wine) once petting age has passed. Current industry practices were then ranked and weighted for welfare importance through an online survey completed by 60 industry stakeholders. This survey identified the most important welfare concerns to be poor social grouping of cubs, an inability for cubs to choose their own environment and retreat from an interaction, a lack of trained and dedicated caretakers, and poor breeding practices. The survey results produced a user-friendly tool to score cubs’ welfare in interaction facilities and also identified current practices that are lacking in welfare consideration.

**Abstract:**

African lion (*Panthera leo*) cubs are extensively used in South Africa in wildlife-interaction tourist activities. Facilities provide close interaction opportunities, but the welfare impacts on the cubs are unclear. A workshop was held with 15 lion-experienced stakeholders, including government officials, nature conservationists, animal welfare organisations, lion breeders, lion handlers, an animal ethologist, wildlife veterinarian, wildlife rehabilitation specialist and an animal rights advocacy group representative. Individual representatives nominated a range of welfare concerns, and 15 were identified for discussion and prioritisation. The leading welfare concern was a lack of governance and regulation within the industry. Participants agreed on nine non-negotiable practices affecting welfare concerns, which included ethical concerns, such as cubs exiting into the ranching industry (farming of lions for hunting) and the bone trade (lions being slaughtered for their bones, which are exported for lion bone wine) once petting age has passed. Welfare concerns representative of current management practices within the lion cub interaction industry were compared for importance using an online adaptive conjoint analysis survey of 60 stakeholders in the industry. The survey identified the most important welfare concerns to be poor social grouping of cubs, an inability for cubs to choose their own environment and retreat from a forced interaction, a lack of trained and dedicated caretakers, and poor breeding practices. The conjoint analysis survey results produced a value model, which can be used as a tool to score cubs’ welfare in interaction facilities, and it identified unacceptable practices lacking welfare consideration.

## 1. Introduction

Animal-based tourism is a multibillion-dollar industry that appears to be increasing [1] with wildlife tourist attractions accounting for 20–40% of all global tourism [2]. It is aimed at viewing or encountering wildlife in a range of locations from free ranging to artificially housed, captive animals [3]. A review of public websites revealed that many (43%) animal-visitor direct interaction opportunities offered to the global tourist market centre around the petting of captive wild animals, with other direct interaction activities being feeding, walking or swimming with the animals, riding them, and attending their performances [4]. Understandably, in developing countries the inclusion of wildlife interactions in tourism activities is actively promoted as a revenue generator [5].

D’Cruze et al. [4] determined in their review that while substantial literature exists on the effects of visitor presence on these wild animals, very little research has determined the effects of direct interaction activities on the animals. According to them, this lack of research has resulted in the current lack of clarity on animal interaction practices. Values of conservation, animal welfare, visitor satisfaction, and profitability are often in conflict with each other [6]. Education, research and conservation are well-known objectives of the modern zoo, but so too is entertainment, with visitors seeking to not only learn about and view the animals from a distance but to also interact with them close up [7]. As a result, interacting with wildlife has become a part of the experience at many modern zoos and aquaria, with the public being willing to pay extra for it [8]. Interacting with wildlife also exists in a commercial environment and is often solely for human recreational purposes [9]. The practice is driven by tourist demand, many of whom find interacting with a wild animal enjoyable [10].

de Mori et al. [11] identified a need for the ethical assessment of such animal interactions, which includes comparing the impact on the animal’s welfare with education and conservation outcomes. Similarly, D’Cruze et al. [5] call for a balance between wildlife protection goals and wildlife ecotourism development. Fernandez et al. [7] suggest that when the effects of animals on zoo visitors and vice versa are considered, then opportunities for increased positive animal visitor interactions can be facilitated, thus supporting the goals of modern zoos which include animal welfare, conservation, education, research and entertainment. Fernandez et al. [7] go on to conclude that when animals are provided more “seeming control” over their interactions within a zoo, then the welfare requirements of the animals can be met.

Moorhouse et al. [2] scored 24 types of wildlife tourist attractions and deemed only six to have positive conservation and welfare impacts. van der Meer, Botman and Eckhardt [12] concurred and considered most wildlife tourist attractions to be about making a profit. Essen, Lindsjö and Berg [1] go so far as to describe animals within animal-based tourism as “laborers in a global capitalist economy where they are conscripted into the service of the tourism industry”. Pressure has been placed on travel companies to stop supporting the industry, and some companies have complied [13].

The use of wild animals in tourism is clearly emotive and this supports the need to scientifically review their welfare, with the aim of understanding exactly how it may be compromised and, more importantly, improved. The latest Five Domains Model [14] has included ‘human–animal interactions’ in its assessment of animal welfare. From this we conclude that human–animal interactions have both the capacity to improve and negatively impact the welfare of animals. This seems obvious when looking at companion animals and perhaps even those used in the farming and production industries, with substantial scientific evidence existing on the effects of stockperson behaviour on these animals [15]. This research will therefore complement, by way of examples, not only the latest and amended fifth domain [14] but all domains by interrogating in detail a species-specific animal use system. This research therefore sets out to evaluate the welfare of African lion cubs used in wildlife-interaction tourism within South Africa. While the study focusses on and makes use of information gleaned from the South African interaction industry, the use of lion cubs in tourist attractions is global, with increased trends in the Middle East to own lion cubs as pets for personal interaction satisfaction [16].

Lion cub interaction encounters are a particularly popular international tourism activity. While exact numbers are unknown, it has been estimated that approximately 1000–10,000 lions are used internationally, with annual visitor numbers being 100,000–500,000 [2]. The extent of cub interaction activities offered to the public in South Africa is also not precisely known, but in 2016 it was estimated that lion cubs were potentially readily available from 297 captive lion breeding facilities [17].

There are approximately 7000 lions in captivity [18], owing to their use in captive breeding and utilization of the species. The types of lion cub interaction activities within this captive industry have not been documented, and not all captive bred cubs are used in lion cub interaction activities. Additionally, not all the cubs that are interacted with go on to be used in other captive lion practices, such as walking with lions, lion hunting and the lion bone trade. A wide range of interaction opportunities exist with lion cubs, which include bottle feeding, picking up and/or stroking of the lion cubs, as well as photographic opportunities. Cubs may also be used in corporate functions, as well as organized school tours both on and off site. Cubs may be interacted with from birth to a maximum of seven months of age; thereafter, they become too boisterous and unsuitable for direct interaction as they pose a potential safety risk to the public.

There were four objectives for this study. The first was to achieve an understanding of the lion cub interaction industry and cub welfare issues associated with the practice, making use of those considered proficient in the field, through a diverse stakeholder workshop. The second was to prioritise the identified welfare issues through assessment of their weight and rank in an online conjoint analysis survey. The results obtained from these two prior objectives informed the third, which was to create a value model that can be used to assess the welfare of lion cubs in such interaction activities. The fourth objective was to ascertain whether workshop and conjoint analysis participant demographics could be associated with opinions and results.

## 2. Materials and Methods

### 2.1. Facilitated Workshop to Identify and Describe Core Welfare Concerns

Recognising that participatory diversity is vital to the success of an expert opinion workshop [19], participants with varying interests and needs were invited and provided the substantive themes of the event and the results. Thirty-three South African stakeholders were initially contacted via email and then by telephone with the invitation to attend. Fifteen participants attended and 18 declined. All invitations that were turned down were due to an inability to attend as a result of work commitments and/or distance from the venue. The participants represented nine stakeholder groups: nature conservationists (*n* = 2), one from an academic institution and one from a non-governmental organization; an animal ethologist (*n* = 1); a wildlife rehabilitator with experience in lion cub rehabilitation (*n* = 1); government organization officials responsible for both wild and captive wildlife legislation on their permissible use and welfare (*n* = 3); representatives of non-governmental animal welfare organizations (*n* = 2); an animal rights advocacy representative (*n* = 1); a wildlife veterinarian with captive lion experience (*n* = 1); lion breeders (*n* = 2) and lion cub handlers (*n* = 2), one with zoo experience and the other from an interaction facility. Because in such a diverse group some participants may be perceived as having greater knowledge or interests and may dominate the proceedings [19], a professional, independent facilitator was employed, as recommended by Phillips and Phillips [20]. The facilitator was experienced in managing events with participatory diversity and possible negative interactions.

The workshop commenced with a welcome and completion of consent forms in which participants could indicate whether their names and/or the organisations that they were representing could be made publicly available. After participants introduced themselves, the objectives of the workshop were explained and a presentation on the meaning of the term “animal welfare” followed to ensure that participants could identify the welfare concerns effectively. This included the definition of welfare (as an animal’s state with regard to the quality and quantity of its experiences) and key principles outlined in Phillips [21].

#### 2.1.1. Identification of Welfare Concerns

Participants were provided with a set of adhesive notes, which were already numbered so that the researchers and facilitator, but not the other participants, could link a stakeholder group (not an individual in the case where more than one was present) with an identified welfare concern. The instructions given to the participants were that they should write one idea per note and that the number of notes allowed was not limited. The purpose of this stage was to gather all the ideas from the group on what they thought the key welfare concerns were within the cub interaction industry. Participants were given as much time as required to do this task. This method of collecting ideas was selected because it ensured that all opinions were represented and that these were not influenced by other participants, which would be expected if only verbal responses were accepted. The facilitator then collected all the adhesive notes and placed them on the wall of the room in clusters with similar themes for inspection by everyone. The stakeholders, assisted by the facilitator, then considered, amended, and agreed upon the grouping of the themes and their descriptions. Stage 1 was completed in two hours.

#### 2.1.2. Identifying and Describing the Core Welfare Concerns

The facilitator asked the participants to consider all the key welfare concerns that had been grouped and identified in Stage 1. Each participant was then given three stickers, which could be used by the researcher and facilitator to identify them. Stakeholders were then instructed to indicate their priorities by placing their three stickers on any three of the welfare themes they considered most important. The votes were then tallied and presented back to the group. Stage 2 was concluded in half an hour.

The identified welfare themes were then discussed to determine practices that commonly and currently take place in the lion cub interaction associated environments in South Africa and to determine levels of welfare concern within each theme. This was done in order to describe and contextualize each of the key welfare concerns identified.

#### 2.1.3. Determining Non-Negotiable Practices

Independent of previous stages, participants collectively discussed and agreed on non-negotiable welfare practices, in terms of activities that violate the welfare of the lion cubs so seriously that they were considered unacceptable and must not occur under any circumstances.

### 2.2. Online Adaptive Conjoint Analysis Survey

An online survey sought the views of a wide range of stakeholders with varied experience of lions and/or lion cubs. Stakeholders were required to be able to categorize themselves as one of the following in order to participate: a South African government official working for a department, especially DAFF (Department of Agriculture, Forestry and Fisheries) or DEA (Department of Environmental Affairs), a nature conservator, an academic with lion research experience, an animal welfare organization representative, an animal rights advocacy group representative, a lion owner and/or breeder, a lion cub handler, a wildlife ethologist, a wildlife veterinarian, or a wildlife rehabilitation specialist.

Known individuals and/or organisations who met this criterion were contacted directly via email and directed to the 1000Minds [22] Internet-based software package. These individuals were also asked to snowball the survey to others whom they, in turn, might know and who they believed would qualify by meeting the survey requirements. This method is known to work well in extending a small and unique cohort of initially invited participants [23], though sampling error is usually impossible to determine. These participants, through their networks, invited other participants who met the eligibility criteria and could potentially contribute to this specific study. The total number of participants invited to participate is therefore not known.

The 1000Minds system applies a method for deriving weights known by the acronym PAPRIKA (Potentially All Pairwise RanKings of all possible Alternatives). Participants were provided with pairs of hypothetical scenarios of current lion cub interaction practices defined by two criteria and were then required to select the scenario that reflected their opinion of the better welfare state for the cubs. Each pair of options had a better welfare criterion linked to a poor welfare criterion. An example is: ‘Select the situation which reflects a cub in a better welfare state, all else being equal (a) a cub is removed from its mother immediately after birth and has less than 100 interactions per day or (b) a cub is removed after two days and is interacted with in excess of 200 times per day or (c) they are equal’. This meant that participants were faced in each scenario with making a trade-off, which allowed for an importance rank to be developed for individuals. Twelve attributes were defined, each with three levels of ranked (lowest to highest) scenarios. This resulted in each participant completing, on average, 98 pairwise ranked questions.

After completing the adaptive conjoint analysis portion of the survey, participants completed a further four demographic questions linking their results to their affiliation, years of experience in that affiliation, their gender and whether they resided in South Africa or not.

### 2.3. Statistical Analysis

#### 2.3.1. Facilitated Workshop to Identify and Describe Core Welfare Concerns

It was hypothesized that there would be a relationship between the key animal welfare concerns raised and the stakeholder group that had prioritised them through their vote. A non-metric multidimensional scaling (NMDS) ordination was run using the Vegan package in R [24] to plot similarities and dissimilarities in their responses.

#### 2.3.2. Online Adaptive Conjoint Analysis Survey

The 1000Minds software summarizes the data and presents relative weightings for each welfare attribute per participant [22]. Demographic information was analysed using GraphPad InStat v. 3.06 [25]. A series of χ^2^ contingency tables analysed the effects of each demographic criterion independently of the mean of the welfare concern weightings. A non-metric multidimensional scaling (NMDS) ordination was run using the Vegan package in R [24] to plot similarities and dissimilarities in their responses. This determined associations between participants’ affiliation and the mean weighted utility value of the attributes they selected.

## 3. Results

### 3.1. Facilitated Workshop to Identify and Describe Core Welfare Concerns

Twelve of the fifteen participants indicated that they did not wish to be anonymous, and that the opinions they expressed, either in their professional and/or personal capacities, should be acknowledged.

#### 3.1.1. Identification of Welfare Concerns

The welfare concerns identified by the 15 stakeholder participants during Stage 1 of the facilitated workshop are listed according to the votes received during Stage 2 of the same facilitated workshop (Table 1). Stakeholder participants who identified the original welfare concerns as well as those who voted for them as important after having all the welfare concerns at their disposal to review are presented.

‘Inadequate governance and regulation’ was identified in Stage 1 by only two groups, the government organisation representatives and nature conservationists, but was then rated to be the leading welfare concern during Stage 2 of the workshop, being raised by six of the nine stakeholder groups. Discussion focused on the lack of knowledge on the size and extent of the industry and the need for registration of facilities, the setting of standards supported by regulation, including compliance procedures, and the training of handlers and caregivers.

All except the animal ethologist and the welfare non-governmental bodies identified nutrition as the next most important welfare concern. Both the quality and the quantity of the food provided were identified as core nutritional concerns, which was believed to lead to poor physiological development. The wildlife rehabilitator further identified incorrect feeding techniques as a welfare concern, such as when inexperienced volunteers and/or interaction participants bottle-feed the cubs, which can lead to aspiration pneumonia if milk enters the lungs.

The amount of time participants spent interacting with the cubs was the third most important concern. Participants reported that cubs need time to rest and institutions may have the cubs on display for long periods. This was perceived to lead to physical and mental developmental problems, adding to future welfare concerns.

Having a lack of choice in their environment was identified next, which chiefly focused on the inability of the cubs to choose to retreat from the constant forced human interactions and seek refuge. Although it was reported that some institutions do have an area to which cubs can retreat, others have cubs permanently on display with constant access during hours open to the public.

Inbreeding in captive lions was identified as a further, but less important, welfare concern for the individuals affected by the associated side effects. It was considered to originate from the desire to purposefully retain a genetic trait (such as a large black mane in males, a desirable trait for the hunting industry, a possible destination for future cubs) but also linked to ineffective record keeping and stud book management, which in turn was related to inadequate governance and regulation.

The stakeholders defined behavioural knowledge as the necessary knowledge of lion cub behaviour required in order to raise and handle the cub correctly. The lack of such knowledge by lion cub handlers was recognised as a welfare problem. Animal welfare organisation representatives raised concerns around methods of training the cubs to ensure safe interactions with participants, particularly the use of negative reinforcement, and the animal ethologist identified imprinting, habituation, and desensitisation of the cubs as welfare-related concerns. A further concern was raised regarding the species-specific needs of the cubs, related to their limited ability to express their natural behaviours within the constraints of the interaction environment.

The impact of forced removal of the cub from its mother was believed to cause welfare problems with the cub whose development was likely to be affected, and the mother who would potentially suffer separation anxiety. These problems may be exacerbated by repeated forced removals. Participants reported that captive breeding mothers often reject their cubs, which was discussed as a further welfare concern.

Exit strategies for these hand-reared cubs was nominated as an issue, relating to where the cubs go when they are too old for cub interaction activities. While no specific welfare concerns were listed, it is speculated that ethical issues pertaining to canned hunting (hunting of a captive large predator or one which is tranquilised and/or lured artificially) and the bone trade were the underlying causes for concern.

Hygiene was cited as a welfare concern for two reasons: first, the hygiene of the housing facility and second, potential zoonotic disease transfer to and from the household pets of interaction participants, who may then convey the disease to the cubs during the interaction.

Sleep time was believed to be compromised by the need for people to interact with the cubs over extended hours. The lack of enough sleep or even the quality of sleep were therefore identified as welfare concerns.

The nomination of ‘age of removal’, as a separate concern to the ‘impact of removal’ concern, was discussed, and the stakeholders believed them to be two separate issues with their own separate welfare concerns. The age of removal specifically referred to whether access was given to colostrum and the mothers’ milk for an adequate time prior to being removed. This welfare concern was therefore mainly linked with the nutrition of the cub. The impact of removal was mentioned as possibly resulting in a psycho-physiological stress.

Another welfare concern nominated was the social needs of cubs, referring to the denial of the social interaction that they normally get from other lions in a pride, with the most pronounced being that from the mother. A specific case was that of isolated cubs on display away from other cubs as this is the only existing bond they have with conspecifics.

The affective state of the cubs was one of the final issues nominated, together with the entry strategy for the cub. It was reported that many cub interaction-offering facilities do not breed some or all of their own replacement cubs, and there is concern for the welfare of both the cubs coming from distant breeding facilities and the welfare of the mothers in those facilities.

#### 3.1.2. Identifying and Describing the Core Welfare Concerns

Those who identified specific welfare concerns during Stage 1 did not always vote for those issues when asked to rank their importance during Stage 2. Stakeholders initially identified welfare concerns based on their experiences and/or frames of reference. Upon having the wide array of welfare concerns presented to them, stakeholders were able to consider all the welfare concerns in relation to each other and were able to vote for them accordingly.

**Figure 1:**
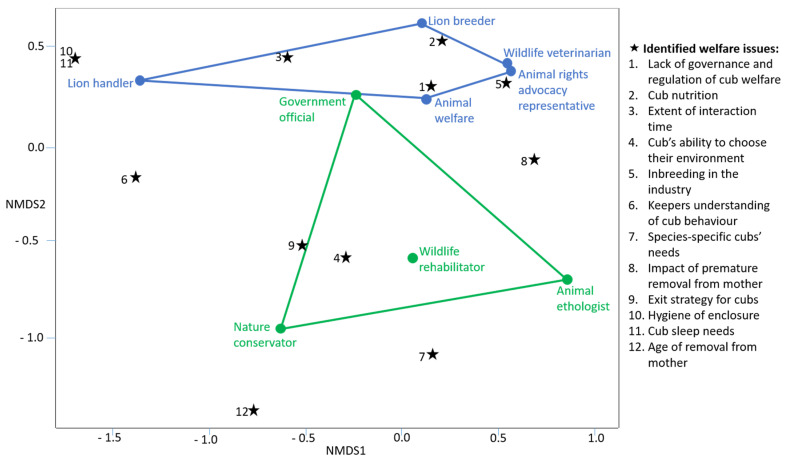
The non-metric multidimensional scaling ordination indicating the associations between the workshop stakeholder participants and the identified animal welfare themes.

The NMDS ordination (Figure 1) indicated that there was an association between two specific clusters of stakeholders. The first was the lion handlers, lion breeders, wildlife veterinarian, animal rights advocacy representative, and the animal welfare organization representatives, who all typically deal directly with captive lions. The associated issues for this cluster dealt with inbreeding, nutrition, interaction time management, and governance. The second cluster comprised government organisation officials, the wildlife rehabilitator, animal ethologist, and nature conservators, whose experiences were mostly with wild lions. The issues they considered most pertinent included the cub’s ability to choose its environment, the exit strategy of the cubs, and behavioural species-specific knowledge held by caretakers.

#### 3.1.3. Determining Non-Negotiable Practices

At the end of the workshop, during Stage 3, all the stakeholder participants conversed and unanimously agreed upon the following nine non-negotiable requirements for cub tourism interaction facilities (with additions in parentheses included for explanation):

1. cubs used in interaction activities should not be sedated
2. no cub should suffer prolonged misery if they are ill (referring to euthanasia)
3. no mutilations, such as declawing, should be allowed
4. no cubs interacted with should exit into the ranching industry
5. no cubs interacted with should exit into the animal parts trade
6. no forced (against the cubs’ will) interactions should be allowed
7. no untrained staff can be allowed to handle cubs
8. lion cub interaction facilities must have access to a wildlife veterinarian
9. no sick animals may be used in public displays

### 3.2. Online Adaptive Conjoint Analysis Survey

Sixty participants completed the online survey and 41 incomplete surveys were discarded because an adaptive conjoint analysis survey requires completion for analysis to be conducted. The demographics of the participants are presented in Table 2. They were from all 10 qualifying affiliations invited, though groups such as nature conservators (25.0%) and government officials from relevant departments (18.3%) dominated the responses. Representation from wildlife veterinarians (10.0%), academics with lion research experience (11.7%) and animal welfare group representatives (11.7%) were similar. Experience in these affiliated fields was dominated by those with 16–20 years of experience (25.0%) and those with 6–10 years (21.7%). Females dominated the participants (65%) and most responses were received by South African residents (81.7%).

**Figure 2:**
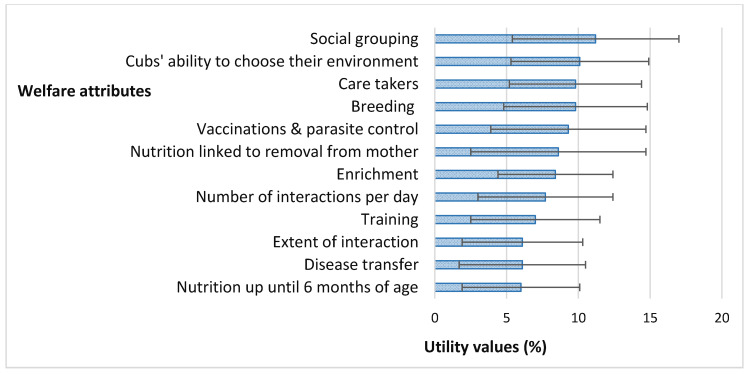
Utility values of the welfare attributes included in the online adaptive conjoint analysis survey, indicating their mean values and standard deviations.

The normalized attribute weights and the mean utility values of the individual levels within the attributes are presented in Table 3. The weight when multiplied with the single attribute score produced the mean utility values for each attribute expressed in Figure 2. Utility values represent the relative importance (weights expressed in %) of the attributes, summarized by the attribute rankings. These utility values ranged from 6.0 to 11.2% and were ranked in the following descending order: social grouping > cubs’ ability to choose their environment > care takers > breeding > vaccinations and parasite control > nutrition linked to removal from mother > enrichment > number of interactions per day > training > extent of interaction > disease transfer > nutrition up until six months of age.

Kendall’s Coefficient of Concordance of the 60 participants’ 24 marginal utility value rankings was 0.126 (range 0–1) which reflects a low level of agreement between participants. A χ^2^ test for independence revealed no significant relationship between the mean weighted attributes and affiliations (χ^2^_88_ = 66.961; *p* = 0.95), years of experience (χ^2^_55_ = 30.128; *p* = 1), gender (χ^2^_11_ = 0.9122; *p* = 1.00), or residency (χ^2^_11_ = 4.866; *p* = 0.94). The validity of the results depends on the assumption that participants met the demographic criteria.

**Figure 3:**
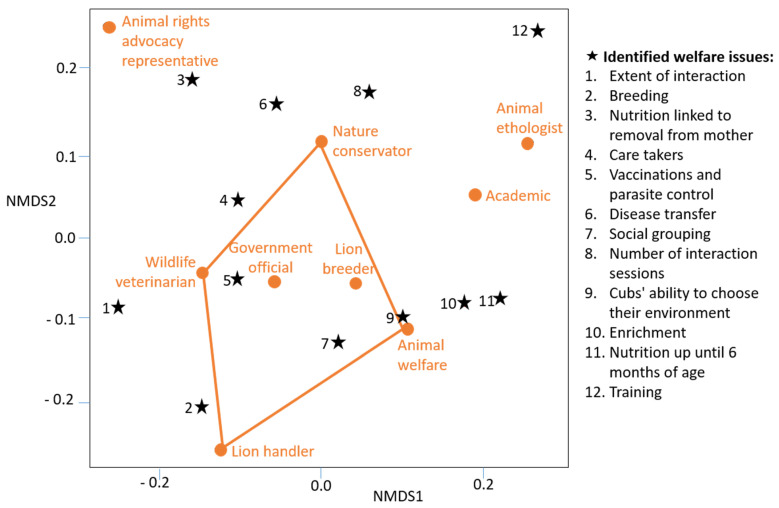
NMDS ordination indicating the associations between the conjoint analysis participants’ affiliation and the mean weighted utility value of the welfare attribute.

The NMDS ordination (Figure 3) indicated that there was an association between the nature conservators, wildlife veterinarians, government officials, lion breeders, animal welfare organisation representatives, and lion handlers in terms of how they voted on the identified welfare concerns. The animal rights advocacy group representatives, academics and animal ethologists appeared as outliers to the other stakeholder groups.

The 1000 Minds software package produces a value model to score a multi-attribute situation [22]. This decision-making tool produces a value for each attribute by multiplying the weight of the attribute with its single attribute score. Within an attribute, the values are relative to each other. Using the ‘Social grouping’ attribute depicted in Table 4 below as an example, ‘Cubs always together’ score a welfare value of 11.2% and this means that it is 3.7% more important to a cub’s overall welfare to be in this state than ‘Cubs grouped but split during interaction hours’, which scored 7.5%. This application does not necessarily provide evidence for the model’s validity [22] but can be used to assist in decision-making. The following derived value model (Table 4) shows how lion cub welfare can be scored at an institution considering the attributes used, calculated from the 60 respondents’ weighted rankings. The score for each attribute selected should be inserted into the score boxes, with the sum of these appearing as the total utility score.

## 4. Discussion

The stakeholder workshop contextualised the welfare concerns that informed the conjoint analysis, resulting in a ranking and weighing of these welfare issues.

### 4.1. Facilitated Workshop to Identify and Describe Core Welfare Concerns

People use many different criteria in judging what constitutes a good life for animals and how animals ought to be treated [26]. Such criteria are informed by frames of reference and experience, and it appeared that the workshop stakeholders exhibited this.

#### 4.1.1. Lack of Governance and Regulation and the Problems of Inbreeding

The lack of governance and regulation in itself is not an actual welfare concern, but the discussion identified that if the extent of the lion cub interaction industry was known and adequately governed, it would be accountable or could be made so for many of the actions affecting welfare. This idea was also identified by Caporale et al. [27] who found effective implementation and enforcement of legislation in Europe to be a vital part of ensuring good animal welfare. The Biodiversity Management Plan for lions (*Panthera leo*) in South Africa [28] identifies the need for a well-managed, captive lion population with developed norms and standards, permit requirements, and mandatory identification of individual animals, including a database with DNA profiles.

Governance and regulation as an animal welfare concern also needs to be extended to persons working with the lion cubs and not only the owners of the facilities. This is important as the cubs would benefit as directly from such regulation. Stakeholders felt that persons directly in contact with the cubs, such as handlers and caregivers, should be required to have some training, as inexperienced handlers and caregivers may cause harm through a lack of knowledge. Inappropriate handler behaviour towards animals can cause fear of humans, which in turn affects their long-term welfare [29]. Lion cubs are in contact with many humans daily, which could influence their health status and welfare. Training and selecting handlers and caregivers to have desirable attitudes and behaviours towards the animals in their care would substantively improve the animals’ welfare [29]. In South Africa, caregivers and handlers at lion interaction facilities are often young adults with very little or no experience at the time of employment, and many are overseas volunteers, who pay to raise and bottle-feed the cubs [30].

Although inbreeding was identified as a lesser animal welfare concern, it can be linked to a lack of governance and regulation. Inbreeding as a welfare concern could be reduced if the objectives laid out in the Biodiversity Management Plan for Lions in South Africa [28] are achieved, especially the introduction of DNA profiling of all lions. There is a rare phenotype or colour variant of the African lion that produces a white coat colour with either yellow, blue, or green eyes. This is a result of a double recessive allele, and therefore white lions are not albinos [31]. Despite white lions only ever occurring naturally in the Timbavati Private Nature Reserve and the adjoining Southern Kruger National Park, they are now commonly found in most captive lion breeding facilities in South Africa. The Association of Zoos and Aquariums [32], in accordance with their Lion Species Survival Plan, discourage the breeding or acquisition of white lions, as all individuals currently found in zoos are severely inbred. Inbreeding can cause defects such as high cub mortality, poor reproductive performance, morbidity, and susceptibility to infectious disease [33].

#### 4.1.2. Nutrition, Impact of Removal from Mother and Age of Removal

The Food and Agriculture Organization of the United Nations (FAO) [34] states that a properly balanced diet and water, supplied in adequate amounts, will avoid physical and psychological suffering from hunger and thirst. This report further explains how correct nutrition is crucial for optimal performance and to sustain optimal fitness. It also explains how, in many cases, it is not only the absence of feed that causes a welfare problem but the feeding of an inappropriate diet. The FAO argues that the disciplines of nutrition and animal behaviour need to be integrated in order to more fully consider the implications of feeding behaviour and nutrition on animal wellbeing. Nutritional requirements are very specific to the age of the cub, and it is essential to consider ‘nutrition’, ‘impact of removal’, and ‘age of removal’ concurrently.

Milk replacer in the absence of mother’s milk must be carefully chosen as cats do not synthesize vitamin D_3_ in the skin and require a dietary source. Hand reared, bottle-fed lion cubs have developed nutritional secondary hyperparathyroidism [35] as a result of having been fed homemade mixtures of cow’s milk and artificial milk blends. The Lion (*Panthera leo*) care manual [32] developed by the Association of Zoos and Aquariums recommends that a formulation based on the mother’s milk is optimal but cautions that the formulation should be supplemented if it does not include taurine [36].

Some facilities wean cubs from a milk replacer bottle-fed diet to commercially available kitten pellets, soaked in milk, followed by pieces of chicken meat, then complete chicken carcasses, followed by chunks of meat (donkey, cattle, and horse) and finally carcass parts. Others wean the cubs from a milk replacer bottle-fed diet to a chicken meat diet and then the entire chicken carcass, with no variety or alternative meat sources thereafter. Sources of chickens may be a concern as they are typically not suitable for human consumption, according to the workshop attendees. Meat (non-chicken varieties) comes from donations of animals that have typically been euthanized as a result of disease or injury where the original owner could not or did not wish to proceed with veterinary treatment. Facilities who choose to feed such carcasses should be aware of the potential hazards associated with potential pharmaceutical drugs administered to the animal prior to euthanasia, pesticide contamination, toxic compounds, and bacteria contaminants [37]. In one such incident, captive cubs were fed a dead pony and developed botulism [38].

The need for careful selection of mineral and vitamin supplements was first demonstrated in the identification of the human disease rickets in lion cubs fed horsemeat in London Zoo in 1889, which was treated by adding goat bones and milk to their diet [39]. Cod liver oil was also recommended for these cubs, since it contains the potentially deficient vitamins A and D_3_, as well as bile salts and taurine, to allow the fat-soluble vitamins to be utilised. A diet restricted to beef meat only is deficient in thiamine, and lion cubs begin to indicate signs of ataxia, general weakness, and seizure like episodes [40]. A Ca:P ratio of 1:1 to 2:1 is required for the normal skeletal development of a lion. Beef and horse meat result in a chronic calcium deficiency due to an excess of phosphorus, as their Ca:P ratios are 1:10 and 1:50, respectively [41]. The side effect of this calcium depletion from the bones results in osteopaenia, muscle pain, and a tendency to develop fractures, which has been reported in captive bred lions [41].

Habituation to humans is central to close and intense encounters, such as is experienced in the interaction industry. Removal from the mother is an inevitable welfare concern that cannot be alleviated as a result of meeting the industry’s needs and objectives. The removal of the cub from the mother potentially causes inadequate nutrition and psychological stress. Farmed mink, for example, are more likely to display abnormal behaviour, such as tail sucking and biting, if they are removed from their mothers at an early age [42]. Striped mice removed from the mother and weaned early were more likely to develop stereotypy behaviour [43]. Early maternal separation in primates results in both behavioural and neurological responses that have been equated with depression in humans [44]. Cubs that exit interaction activities and enter a lifetime of captivity, such as a zoo, may benefit from having been habituated [32], in comparison to a less habituated individual of a more nervous disposition. When removed too early from their mothers, cubs lack the essential colostrum and mother’s milk necessary for healthy development, compounded when poor nutritional milk replacers are used. While predators receive some antibacterial agents via the placenta, these largely come from colostrum, which contains high concentrations of immunoglobulins, leukocytes, and white blood cells [45,46]. Age of weaning of lion cubs from milk replacers varies across facilities, starting from as early as eight weeks of age. In comparison, mother-raised cubs are only weaned at 7–12 months [32].

Tourists at lion cub interaction facilities often ask about the whereabouts of the cub’s mother, and are usually told that she abandoned her cubs [47]. Such statements are for the most part not true, knowing that these cubs are bred to be removed, but in the event that mothers do abandon their cubs, the Lion Care Manual [32] does caution that females should be left totally alone for 24 h, as disturbances in these early stages of rearing may cause the female to neglect or become aggressive towards the cubs. There is also a welfare concern for the mother, and research into mother lion welfare is strongly recommended. Little attention has been paid to the psychological consequences and long-term impacts of breaking the mother–young bond in non-human mammals [48]. The stakeholders questioned the effect of constantly removing cubs from the mother and what would be the most appropriate manner and age to do so. In a zoo situation, “A management strategy that focuses on both maternal care and socialization with human caretakers can provide the best of both worlds. However, this should only be done after careful consideration is given to the temperament of the mother, the experience of the staff, and clear guidelines have been developed for the entire process.” [32]. The same could be said to apply to facilities breeding/sourcing cubs for tourist interaction purposes.

#### 4.1.3. Interaction Time Management, Choice in Environment, Species-Specific Needs, Sleep Needs, Social Needs and Behavioural Knowledge

These were all raised as independent welfare concerns but are discussed together as they all impact upon the behaviour of the lion cubs used in the interaction activities. Interaction time is believed to be highly variable, depending on the many factors that influence visitor numbers and management of the cubs, including the day of the week and the time of year. Some interaction facilities operate from 800–2000 h and the cubs are exposed to a constant flow of interaction participants during this time, with as many as 500 interactions taking place in this period [47]. Other facilities may have peak periods with variable numbers or only operate at set times of the day. Lion cubs naturally spend a very large proportion of the day sleeping, but inactive cubs are less interesting to visitors, which may result in visitors provoking or interacting with the animal on their own terms [7]. Facilities manage the extent of contact with the cubs as they see fit, with some allowing the cub to be carried around whilst others have a more controlled approach, restricting the interaction to a stroke on the back. In the wild, lion cubs naturally engage in extensive play behaviour and visual exploration of their environment [49], but less when their mothers have left to hunt [50]. Similarly, cubs are regularly groomed by their mother under natural conditions [51]. As well as the usual motor play of running, rolling, climbing, and turning, cubs will engage in stalking, ambushing, and grappling—all motor patterns that they would normally use as adults to capture prey [51,52]. The extent of these behaviours will depend on the complexity of their environment and the cognitive stimulation of the cubs. To what extent visitor presence affects these natural behaviours is not yet known.

Waiblinger et al. [53] explain how interactions are perceived by both the humans and the animals used in the interaction. These animals are not just affected by the current interaction taking place but also have residual effects from past interactions. The way in which an animal perceives these interactions can affect its future human–animal relationships [54], potentially resulting in profound consequences on that animal’s life. Negative interaction experiences such as being prodded and pulled will have an immediate negative behavioural response but also affect the way cubs will respond to a future interaction attempt. This implies that what lion cub interaction facilities allow to be done to the cub, within the interaction experience, can have a lasting welfare impact on the cub’s life, either by how they respond to future interactions or even resulting in long-term behavioural problems.

The inability of the cubs to retreat from a forced human interaction and seek refuge was seen as a welfare concern by the stakeholders, but any retreat impacts negatively on the visitors’ experience and desire to interact with the animal they have come to see [7]. This may lead to fewer visitors and less financial gain. Noisy, active crowds are the biggest source of stress [7]. The benefits of providing a retreat space for animals in petting interactions at a zoo mainly lie in allowing for rest and reduction of undesirable behaviours [55] such as aggression towards the interactors. The Lion Care Manual [32] supports this, suggesting that more space and choices about where lions can spend their time may prevent social and behavioural problems.

The African lion’s complex social structure makes them unique among wild cats. The Lion Care Manual [32] requires them to be housed in numbers of sufficient size, so as to meet their social and behavioural needs. Often cub siblings are removed together and housed in interaction facilities together. The welfare plight of a solitary housed cub is therefore assumed to be worse, given it will have no interaction with any other member of its kind. Appropriate social groupings are important to provide examples of species-typical behaviours [56]. This links the species-specific needs of the cubs to social needs, also identified as a welfare concern. Suboptimal group sizes are associated with increased abnormal behaviours in a range of captive mammals [56]. The Lion Care Manual [32] explains how the cub’s exposure to adult lions is a critical component of a cub’s social development, hence, all options (even males) should be considered. It emphasizes that this is especially important for singletons. This social and species-specific requirement cannot be met when cubs are housed separately from adults for interaction purposes.

Stakeholders felt that the behavioural knowledge held by cub handlers and keepers was linked with their training and disciplining of the cubs to ensure that they were safe to have around the interacting public. So called inappropriate behaviour on the part of the cubs requires defining and understanding. Zoos have some influence on keeper skills and behaviour [57], and only positive reinforcement training should be used [58]. The Lion Care Manual [32] encourages a relationship between keepers and lions that is built on trust and positive interactions, resulting in lions that are comfortable and cooperative with people. However, it has been suggested [32] that interactions with cubs in zoos should cease at around three months of age, as it is at this time that the cubs become dangerous. It is not known whether this age may, however, be too late in terms of developmental damage incurred. Many cub interaction facilities in South Africa use cubs for interactions up until six months of age, or for as long as the temperament of the cub allows this. The training required to achieve this state needs to be fully understood and scrutinized from a welfare perspective.

#### 4.1.4. Hygiene

Hygiene was perceived as a welfare concern for two reasons. First, the popularity of open farms and petting zoos has increased over the years and the open access policy of these establishments allows visitors to be in direct contact with animals, which may lead to the transmission of pathogens from animals to humans [59] and potentially from household pets to the cubs through the human interactors and caretakers as the vector. African lions are susceptible to the same diseases as domestic carnivores [60]. Options to control the transmission of diseases are hand washing and sanitizers before and/or after each interaction and vaccinating cubs prior to the initiation of interactions with the public. In the latter case, facilities can only allow interactions to begin when the cubs are eight weeks of age, as this is the earliest at which the vaccines can be administered. Lion cubs should be vaccinated against feline rhinotracheitis (FRV), feline calicivirus (FCV), feline panleukopenia virus (FPLV), and rabies. Parasite control is usually also applied to cubs in facilities that choose to vaccinate. The Lion Care Manual [32] mandates the vaccination of cubs in their facilities, and vaccinations of bred wild carnivores is encouraged by the South Africa Veterinary Association [61].

#### 4.1.5. Entry and Exit Strategies

The entry and exit strategies for lion cubs used in interaction activities were both considered welfare concerns, even though these are events that take place prior to and after the period of activity being evaluated in this study. Reports by animal welfare groups [62] and animal rights advocacy groups [63] indicate that many cubs in South Africa are bred in poor conditions for hunting, and that facilities may use cub interactions as an opportunity to generate additional income from the use of the cub. It could be argued that wherever the cub goes, its life and use should be humane to justify its use in the interaction facility. Current known options available to cubs include sale to a zoo, use in lion-walking safaris up until approximately two years of age, entry into a breeding programme, being hunted and/or entering the lion bone trade. There is little evidence of the fate of most cubs.

#### 4.1.6. Affective State

Finally, the cubs’ affective state was identified as a welfare concern. The stakeholders initially stated wellbeing as a welfare concern and upon being asked to clarify, indicated the affective states of the cubs. This demonstrates the confusion in terminology that exists in some sectors of the industry. For some, wellbeing is a synonym for welfare, used more in the USA to avoid confusion with the welfare state [21]. For others [64], it is the presence of pleasant mental states and the absence of unpleasant ones, i.e., a hedonism that may be difficult to achieve for lion cubs used in an interaction activity.

#### 4.1.7. The NMDS Analysis

This analysis identified a similarity between the nature conservators, wildlife veterinarians, government officials, lion breeders, animal welfare organisation representatives and the lion handlers in their identification of welfare concerns. This suggests a common perspective of those most directly involved in the practices. Those identified as outliers, the animal rights advocacy group representatives, academics, and animal ethologists, are not as closely involved. However, the sample size is small, and it is possible that this difference identified was influenced by personally held beliefs rather than being linked to one of their group memberships, in this case their profession.

The diversity of the nine affiliated stakeholders and likelihood that each would judge welfare differently ensured that lion cub welfare was considered from a broad perspective, rather than a scientific or legislative view alone. This may also explain why general issues, like inadequate governance and regulation, were considered primary welfare considerations, due to their relevance to several stakeholder groups. The suitability of the identified wide range of stakeholder group was proven by their ability to make informed decisions when discussing and voting on welfare concerns. This explains why breeders, the rehabilitator, an animal rights advocate, veterinarian, and animal welfare organisation representatives, who had not initially identified governance and regulation to be an issue, did so after discussions, and then voted it as one of their top three welfare concerns. The commonality of views of the nature conservationists and government officials, who both identified governance and lack of regulations as a key welfare concern, was expected as they both experience issues in this regard when working with the captive lion industry.

### 4.2. Online Adaptive Conjoint Analysis Survey

Limited literature exists on the welfare of lion cubs used in wildlife tourism interactions, therefore this method allowed a wide range of stakeholders with opinions and expertise in their fields to rank identified welfare attributes arising from current management practices as scenarios by using a trade-off methodology. While the software was able to rank these attributes based on their relative mean weightings in percentages, the high variance attained for most attributes and attribute levels reveals that there is very little agreement on their prioritisation across demographic groups.

The NMDS ordination (Figure 3) depicts the associations of the affiliated responses in terms of their mean weightings. The single wildlife rehabilitator was excluded from analysis, as the opinion of only one cannot be representative of an affiliation. The animal rights advocacy representatives are outliers in the ordination, possibly due to their absolutist approach to moral decision-making [65]. The ethologist and lion research academics are associated outliers in another direction but have some association with each other. The applied natural sciences emanating from species-specific behaviours may assist in explaining the association.

The remaining affiliates, namely nature conservators, wildlife veterinarians, government organisations, lion breeders and/or owners, lion cub handlers, and animal welfare group representatives, appear to be associated by their broad fields of involvement that currently (specifically in SA) require them to work closely with each other when it comes to lion cub interactions. The captive lion industry currently employs and/or works with each of these affiliates. Welfare attributes associated with these affiliates include disease transfer, caretakers, breeding, vaccinations and parasite control, social groupings of cubs, and their ability to choose their environment as well as enrichment.

The value model produced by the responses of the 60 participants is a potential tool to be used by an institution or organization to help evaluate the welfare of cubs used in wildlife tourism activities. This tool can also be used to determine where welfare shortfalls exist and allows welfare weights for attributes to be improved, thereby improving the overall welfare of the cubs. The welfare model is limited to the attributes included in the conjoint analysis survey, which have support in the literature. The attribute weightings (%) require validation through scientific evidence such as lion cub behavioural studies; however, the model has strength because it was generated with the participation of a wide range of stakeholders.

When the value model is practically applied, all the lowest level attributes could be seen as representing a welfare deficient situation for the lion cubs. The 12 lowest level attributes can therefore be seen as unacceptable practices from a welfare perspective. Some of these lower-level attributes need to be redefined if deemed unacceptable in order to avoid confusion with middle levels. They are as follows:

1. no forced interactions should be allowed, and cubs should be allowed to retreat from an interaction
2. no untrained staff may be allowed to handle cubs and multiple inexperienced volunteers and visitors may not be responsible for bottle feeding
3. cubs should not be purposefully inbred to retain traits
4. cubs should not be removed from the mother before 2 days of age
5. weaning from milk replacers as young as 2 months and a diet restricted to chicken only is unacceptable
6. interactions must not exceed 200 per day
7. training should be restricted to handlers only and not exceed a tap on the nose
8. cubs should not be raised in isolation
9. cubs should not be carried and handled by interactors
10. parasite control should be mandatory
11. all interactors should wash their hands prior to interacting
12. climbing structures should be provided for the cubs

The lowest level of welfare (0% in the model) is as follows: being purposefully inbred to retain genetic traits, removed from its mother at birth and raised in isolation by multiple inexperienced volunteers and bottle fed by visitors; interacted with over 200 times a day, prevented retreat from an interaction and regularly carried and handled, unwanted cub behaviour is discouraged by handlers and interactors, no toys or climbing structures are provided; replacer milk is only provided until two months of age; diet comprises of chicken only; no vaccinations or parasite control is provided and interactors do not use footbaths or hand disinfectants.

Conversely, the best welfare possible (100% in the model) is as follows: cub is bred making use of a studbook, it remains with its mother up until two weeks of age and is always kept together with its siblings; only handled by trained full time handlers responsible for raising the cubs; no training of the cubs takes place; cub is interacted with less than 100 times a day, is not interacted with when sleeping and may retreat from an interaction; toys and climbing structures are provided for the cub; replacer milk is provided up until 6 months of age while introducing scientifically suitable feline pellets then chicken and meat including carcasses; vaccinations and parasite control is provided and interactors make use of footbaths and hand disinfectants. This best welfare practice just described must be contextualized to the existing lion cub interaction industry. The attainment of a 100% welfare score only implies that the welfare level is as good as it can be within such an industry. It does not mean that welfare cannot be further improved upon, including by the cub not being included in such an industry in the first place.

The importance of addressing all welfare concerns becomes evident when trying to improve the welfare of lion cubs within such an industry. While those concerns with higher weighted ranking scores address a greater welfare need, it is the combination of welfare concerns that determines the overall welfare of the cub. A tool allowing one to assess welfare in a lion cub interaction facility should determine a quantifiable level, below which it is considered unacceptable. A 0% welfare score is clearly not this limit. The next level and value up from the 0% score within each attribute, therefore, represents the minimum welfare requirements, the sum of which is 57%.

## 5. Conclusions

The results revealed that stakeholders are capable of identifying and prioritising welfare concerns outside of their areas of understanding when empowered with a wide scope of knowledge, post expert consultation. Research and policy in such fields should therefore seek to gain insights from as wide an array of stakeholders as is possible. The value model further brings about an understanding that, in order to address welfare in practice, all concerns need to be accommodated, whilst considering their ranked importance. The study design provides an example of how wildlife interactions can be assessed to mitigate welfare concerns that could be useful for other species, such as having photographs taken with koalas, elephant riding, and holding of sloths, all popular tourist wildlife interaction activities.

The non-regulated activity of lion cub interactions is in urgent need of relevant and applicable guidelines within which to operate.

The ethical issues identified through the non-negotiables emanating from the workshop indicate a clear need to distance this specific tourism interaction activity from the possible hunting of the lions and/or their entering into the lion bone trade. The identified and prioritised welfare concerns faced by lion cubs used in interaction activities, guided by a wide array of stakeholder representatives, has resulted in a relevant and user-friendly tool against which to assess the cubs’ welfare. Validation of the model through physiological responses and behavioural studies on lion cubs within such facilities would strengthen its effectiveness in the field.

## Figures and Tables

**Table 1:** Animal welfare concerns identified in Stage 1 and the stakeholders who identified them, listed in declining order of support in Stage 2 (Abbreviations: nature conservationist (NC), government organisation official (GO), wildlife rehabilitator (WR), animal rights advocacy representative (AR), wildlife veterinarian (WV), animal welfare organization representative (AW), lion breeder (LB, lion handler (LH), animal ethologist (AE)).

Identified Animal Welfare Concern	Stakeholder Group Who Identified the Issue in Stage 1	Stakeholder Group Who Voted for the Issue in Stage 2	Number of Votes Cast in Stage 2
Lack of governance and regulation	NC GO	LB GO WR AR WV AW	9
Cub nutrition	LB NC GO LH WR AR WV	LB GO AR WV AW	7
Extent of interaction	GO LH WR AR AW	LB GO LH AW	6
Cubs ability to choose their environment (ability to retreat from an interaction)	LB NC GO	NC GO WR AW	5
Inbreeding in the industry	LB NC GO	AE LB WV	4
Keepers understanding of cub behaviour	AE AW	NC LH	3
Species-specific needs of cubs	AE NC	AE NC WR	3
Impact of premature removal from mother	LB AR WV AW	AE AR AW	3
Exit strategy for cubs (the future of the cub once too old for interactions)	GO WV AW	WR AR AW	2
Hygiene in enclosure	LB GO LH AW	LH	1
Sleep needs of the cubs	LB AR	LH	1
Age of removal from the mother	LH WR AR AW	NC	1
Social needs of the cubs	LB NC		0
Affective states of the cubs	GO AW		0
Entry strategy of the cubs (where the cubs were sourced)	NC GO		0

**Table 2:** Demographics of the online adaptive conjoint analysis survey participants (*n* = 60).

Demographic	No. of Respondents (%)
Affiliated experience:	
Lion cub handlers	2 (3.3)
Lion owners and/or breeders	4 (6.7)
Nature conservators	15 (25.0)
SA government officials working for relevant departments	11 (18.3)
Wildlife ethologists	4 (6.7)
Wildlife rehabilitation specialist	1 (1.7)
Wildlife veterinarian	6 (10.0)
Academics with lion research experience	7 (11.7)
Animal rights advocacy representatives	3 (5.0)
Animal welfare organisation representatives	7 (11.7)
Years of experience:	
<1 year	3 (5.0)
1–5 years	10 (16.7)
6–10 years	13 (21.7)
11–15 years	7 (11.7)
16–20	15 (25.0)
>20 years	12 (20.0)
Gender:	
Females	39 (65.0)
Males	21 (35.0)
South African residents:	
Yes	49 (81.7)
No	11 (18.3)

**Table 3:** The normalized attribute weights and the mean utility values for all the levels within each attribute, with 0 as the least preferred and 100 as the most preferred.

Attribute	Attribute Weight (Sum = 1)	Level	Mean Utility Value
Breeding	0.098	Purposefully inbred to retain traits	0
		Ad hoc breeding	56.4
		Use of a studbook	100
Care takers	0.098	Multiple inexperienced volunteers and bottle fed by visitors	0
		Multiple semi-trained volunteers	55
		Trained full time handlers only	100
Nutrition linked to removal from mother	0.086	Removal after birth	0
		Removal after 2 days	48.5
		Removal after 2 weeks	100
Nutrition up until 6 months of age	0.086	Replacer milk until 2 months followed by chicken pieces then chicken carcasses	0
		Replacer milk until 4 months while introducing chicken pieces then chicken and meat carcasses	61.6
		Replacer milk until 6 months while introducing feline pellets then chicken pieces then chicken and meat carcasses	100
Number of interactions per day	0.077	>200 interactions per day	0
		100–200 interactions per day	44.4
		<100 interactions per day	100
Cubs’ ability to choose their environment	0.101	Sleeping cub interacted with and prevented retreat from an interaction	0
		Sleeping cub interacted with but allowed retreat from an interaction	62.6
		Sleeping cub not disturbed allowed retreat from an interaction	100
Training	0.07	Inappropriate behaviour discouraged by handlers and interactors, as seen fit	0
		Tap on nose for inappropriate behaviour, applied by handler only	60.9
		No training and no repercussions for inappropriate behaviour	100
Social grouping	0.112	Cubs raised in isolation	0
		Cubs grouped but split during interaction hours	67.4
		Cubs always together	100
Extent of interaction	0.061	Carried and handled	0
		Entire body stroked	54.9
		Backs and abdomen only	100
Vaccinations and parasite control	0.093	No vaccinations or parasite control	0
		Parasite control only	51
		Vaccinations and parasite control provided	100
Disease transfer	0.061	No vaccinations or parasite control	0
		Parasite control only	53.8
		Vaccinations and parasite control provided	100
Enrichment	0.084	No toys or climbing structures provided	0
		Climbing structures only	64
		Toys and climbing structures provided	100

**Table 4:** A value model (%) for rating the welfare of lion cubs used in tourism (wildlife interaction facilities).

Attribute	Weighted Ranking (%)	Score Selected
Social grouping		
Cubs raised in isolation	0.00%	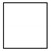
Cubs grouped but split during interaction hours	7.50%	
Cubs always together	11.20%	
Cubs’ ability to choose their environment		
Sleeping cub interacted with and prevented retreat from an interaction	0.00%	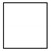
Sleeping cub interacted with but allowed to retreat from an interaction	6.30%	
Sleeping cubs not disturbed and allowed to retreat from an interaction	10.10%	
Care takers		
Multiple inexperienced volunteers and bottle fed by visitors	0.00%	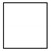
Multiple semi-trained volunteers	5.40%	
Trained full time handlers only	9.80%	
Breeding		
Purposefully inbred to retain traits	0.00%	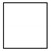
Ad hoc breeding	5.50%	
Use of a studbook	9.80%	
Vaccinations and parasite control		
No vaccinations or parasite control	0.00%	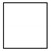
Parasite control only	4.70%	
Vaccinations and parasite control provided	9.30%	
Nutrition linked to removal from mother		
Removal after birth	0.00%	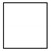
Removal after 2 days	4.10%	
Removal after 2 weeks	8.60%	
Enrichment		
No toys or climbing structures provided	0.00%	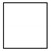
Climbing structures only	5.40%	
Toys and climbing structures provided	8.40%	
Number of interactions per day		
>200 interactions per day	0.00%	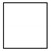
100–200 interactions per day	3.40%	
<100 interactions per day	7.70%	
Training		
Inappropriate behaviour discouraged by handlers and interactors, as seen fit	0.00%	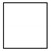
Tap on nose for inappropriate behaviour, applied by handler only	4.30%	
No training and no repercussions for inappropriate behaviour	7.00%	
Extent of interaction		
Carried and handled	0.00%	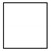
Entire body stroked	3.40%	
Backs and abdomen only	6.10%	
Disease transfer		
No foot baths or hand disinfectants used	0.00%	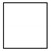
Hand soap wash only	3.30%	
Foot baths and hand disinfectants used	6.10%	
Nutrition up until 6 months of age		
Replacer milk until 2 months followed by chicken pieces then chicken carcasses only	0.00%	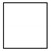
Replacer milk until 4 months while introducing chicken pieces then chicken and meat carcasses	3.70%	
Replacer milk until 6 months while introducing feline pellets then chicken pieces then chicken and meat carcasses	6.00%	
Total utility score (sum of the selected scores) (%)		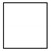

## Data Availability

This research and its data forms part of a PhD thesis registered for with the University of South Africa.

## References

[1] Von Essen E., Lindsjö J., Berg C. (2020). Instagranimal: Animal Welfare and Animal Ethics Challenges of Animal-Based Tourism. Animals.

[2] Moorhouse T.P., Dahlsjö C.A.L., Baker S.E., D’Cruze N.C., Macdonald D.W. (2015). The Customer Isn’t Always Right—Conservation and Animal Welfare Implications of the Increasing Demand for Wildlife Tourism. PLoS ONE.

[3] Newsome D., Dowling R., Moore S. (2005). Wildlife Tourism.

[4] D’Cruze N., Khan S., Carder G., Megson D., Coulthard E., Norrey J., Groves G. (2019). A Global Review of Animal–Visitor Interactions in Modern Zoos and Aquariums and Their Implications for Wild Animal Welfare. Animals.

[5] D’Cruze N., Machado F.C., Matthews N., Balaskas M., Carder G., Richardson V., Vieto R. (2017). A review of wildlife ecotourism in Manaus, Brazil. Nat. Conserv..

[6] Reynolds P.C., Braithwaite D. (2001). Towards a conceptual framework for wildlife tourism. Tour. Manag..

[7] Fernandez E.J., Tamborski M.A., Pickens S.R., Timberlake W. (2009). Animal–visitor interactions in the modern zoo: Conflicts and interventions. Appl. Anim. Behav. Sci..

[8] Kreger M.D., Mench J.A. (1995). Visitor—Animal Interactions at the Zoo. Anthrozoös.

[9] Moorhouse T., D’Cruze N.C., Macdonald D.W. (2017). Unethical use of wildlife in tourism: What’s the problem, who is responsible, and what can be done?. J. Sustain. Tour..

[10] Shani A., Pizam A. (2009). Tourists’ Attitudes toward the Use of Animals in Tourist Attractions. Tour. Anal..

[11] De Mori B., Ferrante L., Florio D., Macchi E., Pollastri I., Normando S. (2019). A Protocol for the Ethical Assessment of Wild Animal-Visitor Interactions (AVIP) Evaluating Animal Welfare, Education, and Conservation Outcomes. Animals.

[12] Van Der Meer E., Botman S., Eckhardt S. (2019). I thought I saw a pussy cat: Portrayal of wild cats in friendly interactions with humans distorts perceptions and encourages interactions with wild cat species. PLoS ONE.

[13] TripAdvisor (2017). TripAdvisor Announces Commitment to Improve Wildlife Welfare Standards in Tourism with Industry-Leading Education Effort and Booking Policy Changes. https://tripadvisor.mediaroom.com/press-releases?item=124874.

[14] Mellor D.J., Beausoleil N.J., Littlewood K.E., McLean A.N., McGreevy P.D., Jones B., Wilkins C. (2020). The 2020 Five Domains Model: Including Human–Animal Interactions in Assessments of Animal Welfare. Animals.

[15] Hemsworth P.H., Coleman G.J. (2011). Human-Livestock Interactions: The Stockperson and the Productivity of Intensively Farmed Animals.

[16] Bachmann A. (2010). Animal Trade in Iraq.

[17] Williams V.L., Sas-Rolfes M.J. (2019). ‘T Born captive: A survey of the lion breeding, keeping and hunting industries in South Africa. PLoS ONE.

[18] Nowark K. (2015). Inside the Grim Lives of Africa’s Captive Lions. https://www.nationalgeographic.com/pages/article/150722-lions-canned-hunting-lion-bone-trade-south-africa-blood-lions-ian-michler.

[19] Mathie A., Greene J.C. (1997). Stakeholder participation in evaluation: How important is diversity?. Eval. Program Plan..

[20] Phillips L.D., Phillips M.C. (1993). Faciliated Work Groups: Theory and Practice. J. Oper. Res. Soc..

[21] Phillips C. (2008). The Welfare of Animals: The Silent Majority.

[22] Hansen P., Ombler F. (2008). A new method for scoring additive multi-attribute value models using pairwise rankings of alternatives. J. Multi-Criteria Decis. Anal..

[23] Morgan D.L., Given L. (2008). Snowball Sampling. The SAGE Encyclopedia of Qualitative Research Methods.

[24] Oksanen J., Blanchet F.G., Kindt R., Legendre P., Minchin P.R., O’Hara R.B., Simpson G.L., Solymos P., Stevens M.H.H., Wagner H. (2014). Vegan: Community Ecology Package. R Package Version 2.2-0. http://CRAN.Rproject.org/package=vegan.

[25] GraphPad InStat (1992). La Jolla California USA: GraphPad Software. https://www.graphpad.com/scientific-software/instat/.

[26] Fraser D., Weary D.M., Pajor E.A., Milligan B.N. (1997). A scientific conception of animal welfare that reflects ethical concerns. Anim. Welf..

[27] Caporale V., Alessandrini B., Dalla P.V., Del S.P. (2005). Global Perspectives on Animal Welfare: Europe.

[28] Funston P.J., Levendal M. (2015). Biodiversity Management Plan for Lion (Panthera Leo) in South Africa. Government Gazette. https://cer.org.za/wp-content/uploads/2010/05/African-lion.pdf.

[29] Hemsworth P.H., Barnett J.L., Coleman G.J. (1993). The human-animal relationship in agriculture and its consequences for the animal. Anim. Welf..

[30] One World 365 (2007–2021) Volunteer with Lions in Africa. http://www.oneworld365.org/animal-volunteer-projects/lions.

[31] Cruikshank K.M., Robinson T.J., Van Heerden J. Inheritance of the white coat colour phenotype in African lions (Panthera leo). Proceedings of the A Symposium on Lions and Leopards as Game Animals.

[32] AZA Lion Species Survival Plan (2012). Lion (Panthera leo) Care Manual.

[33] Trinkel M., Ferguson N., Reid A., Reid C., Somers M., Turelli L., Graf J., Szykman M., Cooper D., Haverman P. (2008). Translocating lions into an inbred lion population in the Hluhluwe-iMfolozi Park, South Africa. Anim. Conserv..

[34] FAO (2012). Impact of Animal Nutrition on Animal Welfare—Expert Consultation 26–30 September 2011.

[35] Van Rensburg I.B., Lowry M.H. (1988). Nutritional secondary hyperparathyroidism in a lion cub. J. South Afr. Vet. Assoc..

[36] National Research Council (2006). Nutrient Requirements of Dogs and Cats.

[37] Harrison T., Harrison S.H., Rumbeiha W.K., Sikarskie J., McClean M. (2006). Surveillance for selected bacterial and toxicologic contaminants in donated carcass meat fed to carnivores. J. Zoo Wildl. Med..

[38] Shamir M.H., Horowitz I., Chaffer M., Grinberg K., Bellaiche M., Elad D. (2008). Botulism in four captive lion cubs; Clinical manifestations and an environmental survey. Isr. J. Vet. Med..

[39] Chesney R.W., Hedberg G. (2010). Metabolic bone disease in lion cubs at the London Zoo in 1889: The original animal model of rickets. J. Biomed. Sci..

[40] Hoover J.P., DiGesualdo C.L. (2005). Blood thiamine values in captive adult African lions (*Panthera leo*). J. Zoo Wildl. Med..

[41] Herz V., Kirberger R. (2004). Nutritional Secondary Hyperparathyroidism in a White Lion Cub (*Panthera leo*), with Concomitant Radiographic Double Cortical Line: Cliical comunication. J. South Afr. Vet. Assoc..

[42] Mason G.J. (1994). Tail-Biting in Mink (*Mustela Vison*) is influenced by Age at Removal from the Mother. Anim. Welf..

[43] Jones M.A., Mason G., Pillay N. (2010). Early social experience influences the development of stereotypic behaviour in captive-born striped mice *Rhabdomys*. Appl. Anim. Behav. Sci..

[44] Gilmer W.S., McKinney W.T. (2003). Early experience and depressive disorders: Human and non-human primate studies. J. Affect. Disord..

[45] Cundiff L.V. (1972). The Role of Maternal Effects in Animal Breeding: VIII. Comparative Aspects of Maternal Effects. J. Anim. Sci..

[46] Chastant-Maillard S., Aggouni C., Albaret A., Fournier A., Mila H. (2017). Canine and feline colostrum. Reprod. Domest. Anim..

[47] Hargreaves R. (2010). Countering the moral and ethical argument for canned hunting of captive bred lions in South Africa. J. WildCat Conserv. Leg. Aid Soc..

[48] Newberry R.C., Swanson J.C. (2008). Implications of breaking mother–young social bonds. Appl. Anim. Behav. Sci..

[49] Ncube S., Ndagurwa H.G.T. (2010). Influence of social upbringing on the activity pattern of captive lion Panthera leo cubs: Benefits of behavior enrichment. Curr. Zool..

[50] Schenkel R. (1966). Play, Exploration and Territoriality in the Wild Lion.

[51] Estes R.D. (2012). The Behavior Guide to African Mammals: Including Hoofed Mammals, Carnivores, Primates.

[52] Bertram B.C. (1978). Pride of Lions.

[53] Waiblinger S., Boivin X., Pedersen V., Tosi M.-V., Janczak A., Visser E.K., Jones R.B. (2006). Assessing the human–animal relationship in farmed species: A critical review. Appl. Anim. Behav. Sci..

[54] Hosey G. (2008). A preliminary model of human–animal relationships in the zoo. Appl. Anim. Behav. Sci..

[55] Anderson U.S., Benne M., Bloomsmith M.A., Maple T.L. (2002). Retreat Space and Human Visitor Density Moderate Undesirable Behavior in Petting Zoo Animals. J. Appl. Anim. Welf. Sci..

[56] Price E.E., Stoinski T.S. (2007). Group size: Determinants in the wild and implications for the captive housing of wild mammals in zoos. Appl. Anim. Behav. Sci..

[57] Hemsworth P. (2003). Human–animal interactions in livestock production. Appl. Anim. Behav. Sci..

[58] Savastano G., Hanson A., McCann C. (2003). The Development of an Operant Conditioning Training Program for New World Primates at the Bronx Zoo. J. Appl. Anim. Welf. Sci..

[59] Stirling J., Griffith M., Dooley J., Goldsmith C.E., Loughrey A., Lowery C.J., McClurg R., McCorry K., McDowell D., McMahon A. (2008). Zoonoses Associated with Petting Farms and Open Zoos. Vector-Borne Zoonotic Dis..

[60] Martella V., Campolo M., Lorusso E., Cavicchio P., Camero M., Bellacicco A.L., Decaro N., Elia G., Greco G., Corrente M. (2007). Norovirus in Captive Lion Cub (Panthera leo). Emerg. Infect. Dis..

[61] South African Veterinary Association (2016). Wildlife. http://www.sava.co.za/2015/10/21/wildlife/.

[62] NSPCA, National Council of SPCAs (2013). Human Interaction with Predator Babies. https://nspca.co.za/predator-cubs/.

[63] (2015). BLOOD LIONSTM. http://www.bloodlions.org/.

[64] Appleby M.C., Sandøe P.T. (2002). Philosophical debate on the nature of well-being: Implications for animal welfare. Anim. Welf..

[65] Galvin S.L., Herzog H.A. (1992). Ethical Ideology, Animal Rights Activism, and Attitudes toward the Treatment of Animals. Ethics Behav..

